# 

**Published:** 1879-08

**Authors:** 


WHOLE NUMBER, CCXVII. AUGUST, 1879. NUMBER 1, VOLUME XIX.

### THE BUFFALO Medical and Surgical Journal.

EDITORS:

THOS. LOTHROP, M. D., A. R. DAVIDSON, M. D.,

P. W. VAN PEYMA, M. D., HERMAN MYNTER, M. D.,

LUCIEN HOWE, M. D.

(Diseases of the Eye and Ear.)

BUFFALO:
PUBLISHED BY THE MEDICAL JOURNAL ASSOCIATION.

**Read the Notice of this Journal among the Advertisements.**

